# Anwendungsbereiche von künstlicher Intelligenz im Kontext von One Health mit Fokus auf antimikrobielle Resistenzen

**DOI:** 10.1007/s00103-023-03707-2

**Published:** 2023-05-04

**Authors:** Christopher Irrgang, Tim Eckmanns, Max v. Kleist, Esther-Maria Antão, Katharina Ladewig, Lothar H. Wieler, Nils Körber

**Affiliations:** 1 Zentrum für Künstliche Intelligenz in der Public Health-Forschung, Robert Koch-Institut, Wildau, Deutschland; 2 FG 37: Nosokomiale Infektionen, Surveillance von Antibiotikaresistenz und -verbrauch, Robert Koch-Institut, Berlin, Deutschland; 3 Fachbereich für Mathematik und Informatik, Freie Universität Berlin, Berlin, Deutschland; 4 P5: Systemmedizin von Infektionskrankheiten, Robert Koch-Institut, Berlin, Deutschland; 5 Robert Koch-Institut, Berlin, Deutschland; 6 Fachgebiet Digital Global Public Health, Hasso-Plattner-Institut, Potsdam, Deutschland

**Keywords:** Künstliche Intelligenz, One Health, Gesellschaft, Gesundheit, Antimikrobielle Resistenz, Artificial intelligence, One Health, Society, Health, Antimicrobial resistance

## Abstract

Die Gesundheit der Menschen steht vor einer Reihe neuer Herausforderungen, die maßgeblich durch den fortschreitenden Klimawandel, den demografischen Wandel und die Globalisierung angetrieben werden. Der One-Health-Ansatz basiert auf dem Verständnis, dass die Gesundheit von Menschen, Tieren und Umwelt eng verknüpft ist. Bei der Umsetzung von One Health in die Praxis ergibt sich die Notwendigkeit, in der Forschung diverse und heterogene Datenströme und -typen aus den verschiedenen Sektoren zu kombinieren und zu analysieren. Verfahren der künstlichen Intelligenz (KI) bieten dabei neue Möglichkeiten zur sektorübergreifenden Beurteilung von heutigen und zukünftigen Gesundheitsgefahren.

Dieser Beitrag gibt einen Überblick über verschiedene Anwendungsbereiche von KI-Verfahren im Zusammenhang mit One Health und zeigt Herausforderungen auf. Am Beispiel der Ausbreitung antimikrobieller Resistenzen (AMR), die eine zunehmende globale Gefahr im One-Health-Kontext darstellt, werden bestehende und zukünftige KI-basierte Lösungsansätze zur Eindämmung und Prävention beschrieben. Diese reichen von neuartiger Arzneientwicklung und personalisierter Therapie über gezieltes Monitoring der Antibiotikanutzung in Tierhaltung und Landwirtschaft bis hin zu einer umfassenden Umwelt-Surveillance für zukünftige AMR-Risikobewertungen.

## Abstract (en)

Societal health is facing a number of new challenges, largely driven by ongoing climate change, demographic ageing, and globalization. The One Health approach links human, animal, and environmental sectors with the goal of achieving a holistic understanding of health in general. To implement this approach, diverse and heterogeneous data streams and types must be combined and analyzed. To this end, artificial intelligence (AI) techniques offer new opportunities for cross-sectoral assessment of current and future health threats. Using the example of antimicrobial resistance as a global threat in the One Health context, we demonstrate potential applications and challenges of AI techniques.

This article provides an overview of different applications of AI techniques in the context of One Health and highlights their challenges. Using the spread of antimicrobial resistance (AMR), an increasing global threat, as an example, existing and future AI-based approaches to AMR containment and prevention are described. These range from novel drug development and personalized therapy, to targeted monitoring of antibiotic use in livestock and agriculture, to comprehensive environmental surveillance.

## Von Public Health zu One Health

**Figure 1:**
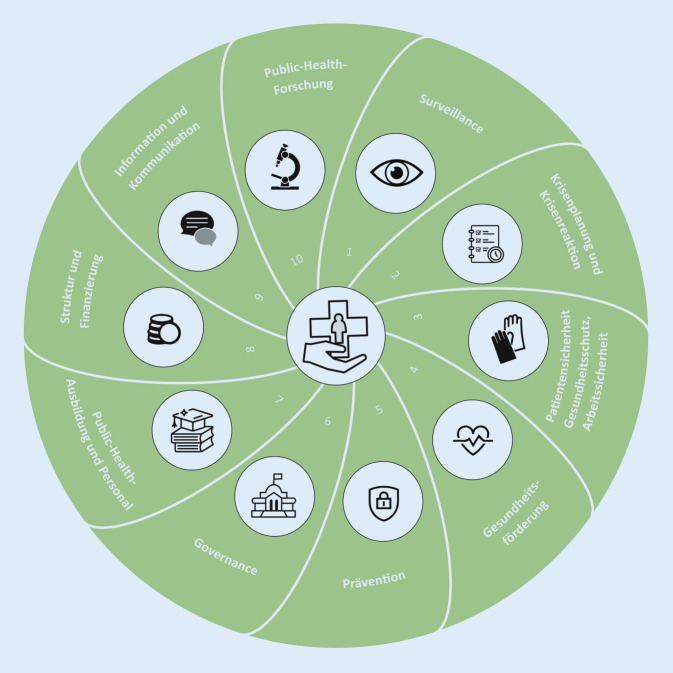

Public Health ist ein systemischer Ansatz zur Erfassung, Sicherstellung und Verbesserung der Gesundheit der Bevölkerung. Die Kernbereiche von Public Health reichen dabei von Surveillance über aktuelle Forschungsfragen, Gesundheitskommunikation bis hin zu Governance- und Präventionsmaßnahmen (Abb. 1). Insgesamt zielen die verschiedenen Kernbereiche darauf ab, den Gesundheitszustand in der Bevölkerung kontinuierlich in allen Gesellschaftsschichten, insbesondere in den sozial benachteiligten, zu verbessern und auf mögliche zukünftige Gesundheitsgefahren vorzubereiten (Resilienz). In Deutschland tragen Public-Health-Strukturen bereits maßgeblich zur Verbreitung eines allgemeinen Gesundheitsbewusstseins bei [1]. Gleichzeitig steht die Menschheit durch ihren globalen Einfluss auf die Umwelt, die Tierwelt und das Klima der Erde vor ganz neuen sozialen, gesundheitlichen und ökonomischen Herausforderungen. Dieser globale und tiefgreifende Einfluss der Menschheit prägt ein neues Erdzeitalter, für das sich die Bezeichnung „Anthropozän“ etabliert hat [2].

Um die bestehenden und neu auftretenden Anforderungen an eine hinsichtlich gesundheitlicher Gefahren resiliente Bevölkerung zu integrieren, muss der Ansatz Public Health breiter gedacht werden. Erweiterungen des bisherigen Verständnisses zielen auf eine möglichst holistische Sichtweise ab, die auch die äußeren Einflüsse aus Tier- und Umwelt einbezieht, welche teilweise vom Menschen mitverursacht werden. Drei Betrachtungsweisen sind One Health, Eco Health und Planetary Health [3]. Namentlich und inhaltlich teilen sich diese Begriffe die Vision, ein globales Gleichgewicht zwischen Mensch, Tier- und Umwelt zu schaffen. Inhaltliche Unterschiede in der Definition globaler Gesundheit und in der Fokussierung auf die drei Sektoren belegen allerdings auch die Komplexität der möglichen und notwendigen Maßnahmen für diese gemeinsame Vision [4–6].

In diesem Artikel stützen wir uns auf die Definition von One Health der Weltgesundheitsorganisation (WHO)^1^: ein integrativer, multisektoraler und interdisziplinärer Ansatz für ein global resilientes Gesundheitssystem durch agile Forschung, Politik und Umsetzungsprogramme. Das Verhalten und die Verhältnisse der Menschen, Tier- und Pflanzenwelt in einem sich durch den Klimawandel verändernden Lebensraum stehen im Zentrum dieses Ansatzes.

Der One-Health-Ansatz hat zusammen mit der laufenden Forschung und den täglich wachsenden Datenströmen innerhalb der einzelnen Sektoren zur Identifizierung neu entstehender globaler und regionaler Gesundheitsgefahren geführt, die sich durch klima- und umweltbedingte Einflüsse in Zukunft weiter verschärfen können [7]. Die Implementierung umfassender Surveillance-Systeme in den verschiedenen One-Health-Sektoren trägt maßgeblich zu neuen Einsichten bei [8]. Bestehende Surveillance-Systeme versuchen, die vielfältigen Dynamiken innerhalb der Sektoren abzubilden, um Gesundheitsgefahren schnell und gezielt zu erkennen [9]. Dazu gehören beispielsweise die klimawandelbedingten Dynamiken bei der Verbreitung von Zoonosen, das zunehmende Auftreten tropischer Infektionskrankheiten in mittleren Breiten [10–12] und die sich im europäischen Raum ausbreitenden antimikrobiellen Resistenzen (AMR).

Durch die stetig wachsenden Datenströme der One-Health-Forschung und -Surveillance ergeben sich diverse neue Anwendungsmöglichkeiten von künstlicher Intelligenz (KI) und maschinellem Lernen (ML; [13]). Im Folgenden stellen wir dar, wie KI die Gesundheits- und Resilienzforschung unterstützen kann. Dafür wird zunächst der prinzipielle Nutzen von KI im One-Health-Kontext aufgezeigt. Als konkretes Beispiel für neuartige KI-Anwendungen richten wir den Fokus auf die Ausbreitung von antimikrobiellen Resistenzen und ihre Prävention. Abschließend benennen wir aktuelle Hürden und Limitierungen für KI in diesem Bereich.

## Künstliche Intelligenz in der One-Health-Forschung

**Figure 2:**
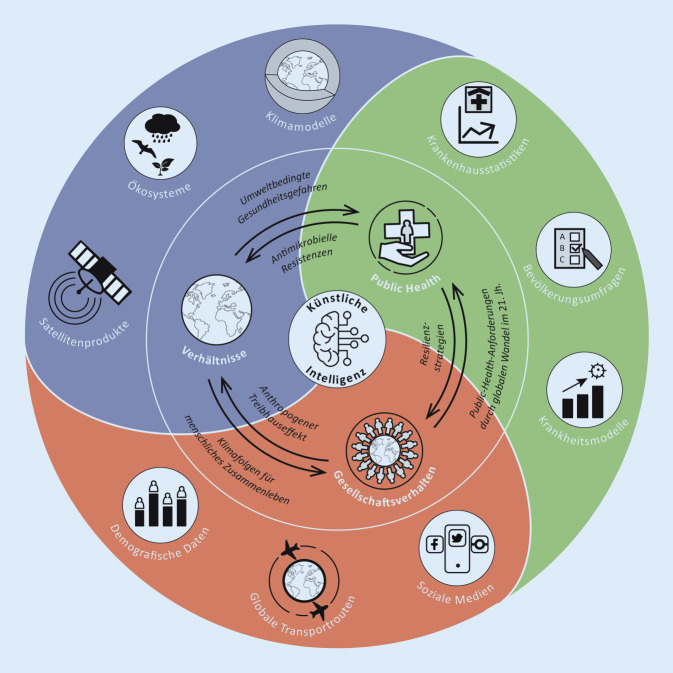

Künstliche Intelligenz (KI) ist ein weit gefasster Überbegriff für lernfähige algorithmische Strukturen, die intelligentes Verhalten zur Lösung von Problemen imitieren und automatisieren können [14]. KI umfasst dabei eine Vielzahl unterschiedlicher Methoden des maschinellen Lernens und ist zu einem festen Bestandteil in der grundlagen- und anwendungsbasierten Forschung sowie in der Industrie geworden. Ein maßgeblicher Unterschied zwischen ML und klassischen Ansätzen der Datenanalyse, Prozessmodellierung und -vorhersage ist die Möglichkeit, Strukturen, Muster und korrelative Zusammenhänge ohne merkmalsspezifische *A‑priori-*Annahmen in problemspezifischen Daten zu suchen und abzubilden. Diese Universaleigenschaft erlaubt es, ML-Methoden gleichermaßen in der Gesundheits‑, Umwelt‑/Klima- und Bevölkerungsforschung einzusetzen (Abb. 2). Die Qualität eines ML-Modells hängt dabei in großem Maße von den zugrunde liegenden Trainingsdaten ab. Da diese nur ein imperfektes Abbild der Realität darstellen, kann es zu verzerrten Modellvorhersagen kommen (Bias). Deshalb ist die Datenerzeugung und -kuration von großer Bedeutung für die Entwicklung von aussagekräftigen Modellen.

Aktuelle Studien demonstrieren den Nutzen von ML zum Beispiel für die abwasserbasierte Epidemiologie zur Überwachung und Detektion von Krankheitsausbrüchen [15], für hochauflösende Wettervorhersagen [16], zur Rekonstruktion von Klimadaten [17] oder zur Erkennung gezielter Desinformation („Infodemie“) in sozialen Medien während der COVID-19-Pandemie [18]. Insgesamt lässt sich ein Großteil der aktuellen KI-Forschung zur Förderung globaler Gesundheit in diese methodischen Schwerpunkte Diagnose, Risikoabschätzung, Überwachung und Vorhersage von Krankheitsausbrüchen und Strategieplanung einteilen [19].

Aktuell werden vorrangig die Potenziale und Grenzen von ML in diesen Bereichen im Rahmen von Pilotprojekten und akademischen Fragestellungen untersucht. Zukünftig wird die operationelle Umsetzung von ML im Hinblick auf One Health und die Feststellung möglicher Effekte auf soziale Determinanten der Gesundheit und die Ziele für nachhaltige Entwicklung der WHO umso wichtiger sein [20].

Für die Entwicklung globaler und planetarer Gesundheit mit dem One-Health-Ansatz kann ML durch die zuvor beschriebene Universaleigenschaft einen maßgeblichen Beitrag leisten. In jedem Sektor von One Health – Menschen, Umwelt, Tierwelt – existieren Datenströme, die jeweilige Variablen, Prozesse und Zusammenhänge aufzeichnen und quantitativ beschreiben. Bei sorgfältiger Erhebung und Kuration der Daten ergeben sich somit auch für ML-Methoden laufend bessere Bedingungen zur umfassenden Abbildung von Prozessen, Diagnosemöglichkeiten und Vorhersagefähigkeiten [13, 21]. Bisher beschränken sich Analysen größtenteils auf einzelne Sektoren, die zukünftige Chance sowie Herausforderung besteht in der Entwicklung von sektorübergreifenden Lösungen.

Zunehmend komplementäre Datenquellen ermöglichen auch die Analyse sektorübergreifender Prozessketten und Kopplungsmechanismen. Sie sind ein zentraler Baustein für die Forschung im Bereich One Health. Hier werden unter anderem die unmittelbaren Auswirkungen der globalen Erwärmung auf die Gesundheit der Bevölkerung bereits intensiv untersucht, zum Beispiel im Hinblick auf: hitzebedingte Herz- und Kreislaufstörungen sowie Todesfälle [22], Ausbrüche von Infektionskrankheiten durch klimabedingte Verschiebung von Vektorhabitaten und zunehmende Globalisierung [23] oder Auswirkungen auf die mentale Gesundheit [24]. Hinzu kommen Forschungsfelder, die weitaus subtilere gesundheitsrelevante Prozesse beinhalten, wie zum Beispiel: mögliche Veränderungen des Malariaaufkommens durch beschleunigtes Abschmelzen des grönländischen Eisschildes [25] und durch Abholzung des Amazonas-Regenwaldes [26], die zunehmenden mentalen Belastungen durch fortschreitende Urbanisierung und durch den Verlust von Ökosystemen [27], sich verändernde Infektionsdynamiken durch Umweltstress, z. B. durch neue Migrationsrouten in der Tierwelt, neue Wirt-Erreger-Interaktionen [28] oder sich ausbreitende klima- und umweltbedingte antimikrobielle Resistenzen [29].

Während sich die Hinweise auf verschiedenste multikausale Prozesse im One-Health-Komplex verdichten, fehlen genaue Quantifizierungen und gesellschaftliche Folgenabschätzungen bisher weitestgehend. ML wird an dieser Stelle in Zukunft als Schnittstellentechnologie eingesetzt werden (Abb. 2) und somit eine Schlüsselrolle für neues Prozessverständnis und letztendlich für die Bildung gesellschaftlicher Resilienz einnehmen. Ziel ist es, durch ML-Verfahren heterogene und unstrukturierte Daten aus verschiedenen Quellen miteinander zu verknüpfen. Dies ist insbesondere dann von Vorteil, wenn die den gesammelten Daten zugrunde liegenden Dynamiken unbekannt sind oder *a priori* nur unzureichend durch bekannte Modelle und Statistiken erklärt werden können.

## Gesundheitsgefährdung durch antimikrobielle Resistenzen (AMR) im One-Health-Kontext

Die Entdeckung und Verwendung von Antibiotika als Arzneimittel in der ersten Hälfte des 20. Jahrhunderts war ein Meilenstein in der medizinischen Forschung. Der medizinische Einsatz hat unzählige Menschenleben gerettet und die durchschnittliche Lebenserwartung erhöht. Verfahren wie Organtransplantationen, große chirurgische Operationen und intensive Chemotherapien in der Tumorbehandlung wurden dadurch erst möglich [30, 31]. Seit Mitte des 20. Jahrhunderts werden Antibiotika neben dem großflächigen Einsatz im humanen Bereich auch vermehrt in der Tiermast eingesetzt sowie zur veterinärmedizinischen Behandlung zahlreicher Infektionen, auch in der Landwirtschaft. Beispielsweise registrieren die US-amerikanischen *Centers for Disease Control and Prevention* (CDC) in den USA jährlich über 600 Antibiotikaverschreibungen pro 1000 Patientinnen und Patienten [32]. Der massive Einsatz von Antibiotika, international und auch in Deutschland, hat durch natürliche Selektion die Ausbildung von Resistenzen gefördert, die eine globale Bedrohung darstellen [33, 34].

Im Jahr 2019 wurde weltweit der Tod von fast 5 Mio. Menschen mit Antibiotikaresistenzen assoziiert, wobei 1,27 Mio. Todesfälle direkt auf die Antibiotikaresistenz der Keime zurückzuführen sind, womit sie schon jetzt die dritthäufigste Todesursache darstellen [32]. Dabei sind Antibiotikaresistenzen nur ein Teilaspekt einer generellen antimikrobiellen Resistenz, die neben Bakterien auch Viren (antivirale Resistenz), Pilze (Antimykotika-Resistenz) und Parasiten (Antiparasitika-Resistenz) umfasst. Die WHO hat erst kürzlich den ersten Report zu gesundheitsgefährdenden resistenten Pilzen veröffentlicht [35]. Pilzinfektionen betreffen weltweit mehr als eine Milliarde Menschen, weshalb die Gefahr durch zunehmende Resistenzen keinesfalls unterschätzt werden sollte [36].

Antimikrobielle Resistenzen stellen eine globale Herausforderung dar, der nur mit einem entschiedenen Umdenken in einem One-Health-Kontext begegnet werden kann [37]. Die universelle Verbreitung von Mikroben bei Menschen und Tieren sowie in der Umwelt, ihre Interaktion untereinander und mit der Umgebung sowie ihre Fähigkeit, Resistenzen durch Selektion auszubilden, machen eine klare Trennung der einzelnen Bereiche unmöglich.

Massentierhaltung [38] sowie die übermäßige Verschreibung von Antibiotika fördern das Problem der Antibiotikaresistenzen gleich mehrfach. Durch unsachgemäße und prophylaktische Verschreibung, falsche Dosierung sowie Fehler bei der Einnahme kann die Bildung von Resistenzen gefördert werden. Allerdings werden die Mechanismen, aufgrund derer eine unsachgemäße Behandlung zur Entstehung und möglicherweise Verbreitung von Antibiotikaresistenzen beitragen kann, noch unzureichend verstanden [39]. Die Belastung der Umwelt mit Antibiotika [40] könnte die Vermehrung und Verbreitung von resistenten Erregern begünstigen, die anschließend Menschen und Tiere befallen können. Dies kann sowohl die direkte Konsequenz eines antibiotikavermittelten Selektionsdrucks sein als auch durch eine Störung des mikrobiellen Gleichgewichts hervorgerufen werden.

In Krankenhäusern können sich resistente Infektionserreger besonders gut ausbreiten, da vulnerable Patientinnen und Patienten bei ungenügender Hygiene einem höheren Infektionsdruck ausgesetzt sein können. Darum ist die Krankenhaushygiene von besonderer Bedeutung. Darüber hinaus gelangen die Medikamentenreste und Ausscheidungen in das Abwasser. In einer groß angelegten Studie konnten in Europa von 53 getesteten Antibiotika 17 in unterschiedlichen Konzentrationen im Abwasser nachgewiesen werden [41]. Im nährstoffreichen Abwasser und Klärschlamm herrscht in der Regel eine hohe Bakteriendichte, in der es zum horizontalen Gentransfer von Resistenzgenen kommen kann [42]. Die Ausbildung der Resistenzen wird durch die gleichzeitige Präsenz von Bioziden, Schwermetallen und Antibiotikamischungen zusätzlich verstärkt, wodurch der Selektionsdruck weiter erhöht wird [42]. Über die Kläranlagen gelangen die Antibiotika und antibiotikaresistenten Erreger in Böden und Gewässer. Von dort können sie wiederum über die Nahrung von Tieren und Menschen aufgenommen werden und tragen so weiter zur schleichenden Resistenzbildung bei. Die Umwelt insgesamt bildet dabei ein Reservoir für antimikrobielle Resistenzen, das kontinuierlich wächst [43]. Die stärkste Belastung für die Umwelt geht dabei von Gesundheitseinrichtungen wie Krankenhäusern und Produktionsstätten von Antibiotika aus. Genaue Zahlen zur Verbreitung und Ursache von resistenten Keimen in der Umwelt existieren allerdings nicht.

Eine zusätzliche Verunreinigung der Umwelt durch antimikrobielle Substanzen entsteht durch den großflächigen Einsatz vor allem in der konventionellen Nutztierhaltung. Bei konventioneller Tierhaltung auf engem Raum kann eine Infektion in der Regel nur eingedämmt werden, wenn die gesamte Population medikamentös behandelt wird. Das führt zur Behandlung auch von nichtinfizierten Tieren und fördert somit die unnötige Ausbildung von Resistenzen. Die Gülle der behandelten Tiere wird in der Landwirtschaft zur Düngung der Felder verwendet und antimikrobielle Substanzen gelangen so ins Grundwasser und die Nahrungsmittelproduktion.

Die Umwelt selbst hat darüber hinaus eine rückkoppelnde Wirkung auf antimikrobielle Resistenzen. So existieren starke Hinweise eines Zusammenhangs von Klimafaktoren und der Verbreitung und Ausbildung von antimikrobiellen Resistenzen [29]. Die klimatischen Bedingungen können dabei ganz unterschiedliche Auswirkungen auf die Bildung von antimikrobiellen Resistenzen haben. Durch Änderung des Klimas kommt es zur Veränderung von Lebensräumen und Erschließung neuer Nahrungsquellen, die Austrocknung von Feuchtgebieten führt zu veränderten Verhaltensweisen und die Population von Mikroben wird durch klimatische Bedingungen verändert. Ferner werden das soziale Verhalten von Tieren und Menschen sowie viele weitere Faktoren direkt oder indirekt durch das Klima beeinflusst. Vor allem vektorübertragene Krankheiten, wie Malaria, Dengue-Fieber oder Lyme-Borreliose, können sich durch veränderte klimatische Bedingungen schnell verbreiten. Neben der Gefahr von Resistenzen des Erregers spielt dabei auch die potenzielle Resistenz des Überträgers vor allem gegen Insektizide eine entscheidende Rolle [11, 44]. Der erhöhte Einsatz von Insektiziden durch die rasche Verbreitung von Insekten als Folge des Klimawandels birgt das Potenzial der Ausbreitung von Resistenzen. Umweltfaktoren zusammen mit dem übermäßigen Einsatz von antimikrobiellen Substanzen führen zu einer Zuspitzung der Gefahr durch antimikrobielle Resistenzen.

## KI-Methoden im Kampf gegen AMR

**Figure 3:**
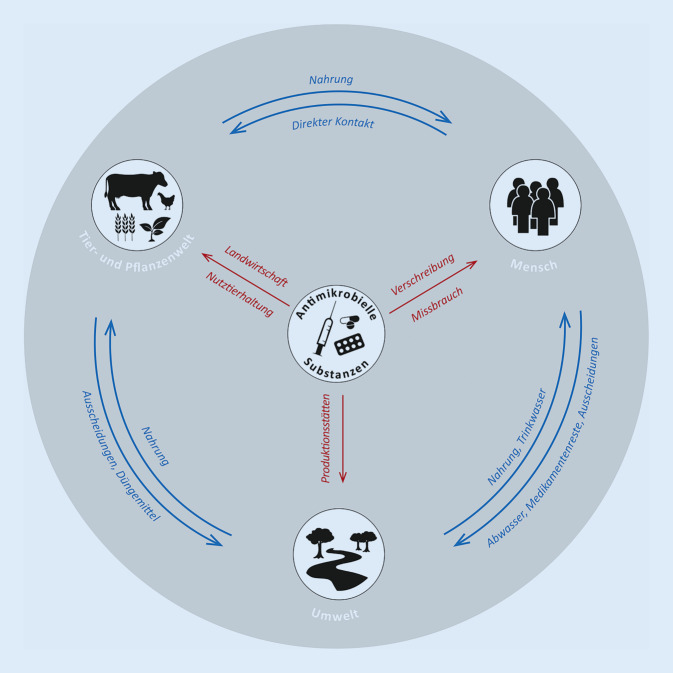

Zur Bekämpfung von antimikrobiellen Resistenzen ist es notwendig, auf mehreren Ebenen Maßnahmen zur Eindämmung zu ergreifen. Moderne KI-Verfahren können dabei von großem Nutzen sein, indem sie helfen die Verbreitung von Resistenzen nachzuvollziehen (Abb. 3; [45]).

Die Erfassung von klinischen Daten kann dabei helfen, Medikamente gezielt zu verschreiben und einen gefährlichen, übermäßigen Einsatz zu vermeiden [46]. Eine personalisierte Medizin optimiert nicht nur die individuelle Versorgung und schont Ressourcen, sondern hilft auch, Probleme, die durch multiresistente Keime entstehen, einzudämmen. Der Einsatz von sequenzbasierten ML-Verfahren kann genutzt werden, um resistente Keime zu identifizieren und die Verschreibung von Medikamenten entsprechend anzupassen [47, 48]. Dieser Ansatz ist beispielsweise seit einigen Jahren etabliert, um eine initiale Therapie gegen das humane Immunodefizienz-Virus (HIV) auszuwählen. Dabei wird häufig auf eine Genotypisierung des Erregers zurückgegriffen mit anschließender ML-basierter Therapieempfehlung. Ähnliche Methoden erlauben die Erstellung eines Therapieplans, der Resistenzentstehung durch antivirale Behandlung verringert [49]. Im Kontext der Anpassung von RNA-basierten Impfstoffen im Kampf gegen SARS-CoV‑2 hat sich die genomische Surveillance bewährt [50], die zusammen mit einer Reihe von am Robert Koch-Institut entwickelten ML-basierten Tools [51] die schnelle Bewertung aufkommender Varianten hinsichtlich eines möglichen *Vakzin-Escape* ermöglicht.

Im Bereich der bakteriellen Erreger finden neben phänotypischen Nachweismethoden immer mehr genotypische Verfahren Anwendung, die Resistenzgene sowie -plasmide nachweisen, sodass antimikrobielle Resistenzen zukünftig mittels ML-Verfahren vorhergesagt werden könnten. Zusammen mit immer schnelleren und günstigeren Genomsequenzierverfahren könnten diese Entwicklungen dazu beitragen, den gezielten Einsatz von Antibiotika besser zu steuern.

Die Suche nach neuen Therapeutika gegen mikrobielle Erreger kann durch ML-Verfahren vereinfacht werden. So werden Algorithmen entwickelt, um die Suche nach Antibiotika zu beschleunigen [52, 53]. Dabei können ML-Verfahren vor allem dabei unterstützen, aus Hunderten Millionen von chemischen Substanzen diejenigen zu identifizieren, die eine antimikrobielle Wirkung haben könnten [54]. Die mikrobielle Wirksamkeit der vorhergesagten Kandidaten wird dann experimentell validiert oder widerlegt. Auf diese Art lässt sich die Suche nach der „Nadel im Heuhaufen“ der chemischen Substanzen stark eingrenzen und minimiert den experimentellen Aufwand. Des Weiteren werden Algorithmen verwendet, um Kombinationen von Medikamenten zu identifizieren, die einen synergistischen Effekt haben, wodurch die Menge der eingesetzten Therapeutika reduziert werden kann [55]. Dabei könnten gezielt solche Kombinationen identifiziert werden, die antimikrobielle Resistenzen verhindern [56, 57].

Algorithmen können dabei helfen, den Einsatz von antimikrobiellen Substanzen in der Nutztierhaltung zu begrenzen. Für den individuellen Einsatz der Therapeutika können ML-gestützte Monitoringsysteme eingesetzt werden, die den Gesundheitszustand der Individuen anhand kontinuierlich gesammelter Gesundheitsparameter überwachen. Dadurch können im Falle einer Infektion die betroffenen Tiere frühzeitig isoliert werden. So wird eine gezielte Behandlung ermöglicht, die Ausbreitung der Infektion begrenzt und die Behandlung unbetroffener Tiere vermieden (Metaphylaxe).

Zur Bekämpfung von antimikrobiellen Resistenzen in der Umwelt ist es zunächst erforderlich, zu erheben, wo Resistenzen bestehen, wie sie sich verbreiten und wo eine hohe Belastung durch antimikrobielle Substanzen vorliegt. Dazu könnte systematisch erhoben werden, in welchen Ökosystemen sich antimikrobielle Substanzen ansammeln und was die Ursachen dafür sind. ML-Verfahren können hier wiederum für automatisierte Analysen von erhobenen Datenreihen und Zeitserien genutzt werden und zu einer entsprechenden Risikobewertung beitragen. Ebenso lassen sich räumliche Muster mit ML-Verfahren untersuchen, um lokale Datenerhebungen miteinander zu verbinden und sie auf nationale oder internationale Ebene zu extrapolieren. Ein solches Monitoring in der Umwelt könnte dabei helfen, Resistenzen frühzeitig zu erkennen und entsprechend gegenzusteuern. Die genaue Erfassung ermöglicht dann auch den Eingriff mittels regulatorischer Maßnahmen in einer bestimmten Region. So könnte beispielsweise temporär und regional der Einsatz eines bestimmten Antibiotikums untersagt werden.

## Herausforderungen und Ausblick

Auch wenn die Möglichkeiten des Einsatzes von KI-Verfahren zur Bewältigung von antimikrobiellen Resistenzen zahlreich sind, so bleiben der konsequente Einsatz und die Entwicklung von Algorithmen zur Bewältigung der Krise aktuell noch hinter den Erwartungen zurück [58]. Für eine Reduzierung von antimikrobiellen Resistenzen ist eine sektorübergreifende Zusammenarbeit notwendig. Innerhalb Deutschlands gibt es verschiedene Kompetenzen und Zuständigkeiten im Rahmen von One Health. Als zentrale Umweltbehörde obliegt dem Umweltbundesamt (UBA) die Beurteilung der Situation in der Umwelt. Für Nahrungsmittel und die Tiergesundheit sind das Bundesinstitut für Risikobewertung (BfR), das Bundesamt für Verbraucherschutz und Lebensmittelsicherheit (BVL) und das Friedrich-Loeffler-Institut (FLI) zuständig. Das Robert Koch-Institut (RKI) beschäftigt sich mit Fragen der öffentlichen Gesundheit und das Bundesinstitut für Arzneimittel und Medizinprodukte (BfArM) sowie das Paul-Ehrlich-Institut (PEI) sind für die Beurteilung und Zulassung von Arzneimitteln zuständig. Eine Zusammenarbeit der Behörden und Institute erfordert, das Daten nach dem FAIR-Prinzip (Auffindbarkeit, Zugänglichkeit, Interoperabilität und Wiederverwendbarkeit) veröffentlicht und zugänglich gemacht werden. Darüber hinaus sollten Schnittstellen und Standards sowie ein rechtlicher und ethischer Rahmen geschaffen werden, die den Austausch von Daten und Algorithmen ermöglichen. Die Bundesregierung hat zur Förderung und Entwicklung dieser Zusammenarbeit die Deutsche Antibiotika-Resistenzstrategie „DART 2020“ erarbeitet. Am RKI werden beispielsweise die Projekte ARS (Antibiotika-Resistenz-Surveillance), AVS (Antibiotika-Verbrauchs-Surveillance) und ARVIA (ARS und AVS – Integrierte Analyse) umgesetzt, um Antibiotikaresistenzen zu überwachen und bewerten zu können. Erste Ansätze einer Integration der Surveillance der verschiedenen Sektoren erfolgte über die German One Health Initiative (GOHI). Neben der nationalen Zusammenarbeit erfordert der stete Personen- und Warenverkehr auch internationale Kooperation mit nationalen und internationalen Behörden wie der WHO.

Zur konsequenten Bekämpfung der Gefahr durch antimikrobielle Resistenzen bedarf es einer gemeinsamen Kraftanstrengung. Eine systematische Erhebung der Daten ist notwendig, die ein Monitoring von Resistenzen sowie den Einsatz von antimikrobiellen Substanzen in den einzelnen Sektoren abbildet. Dazu müssen Verschreibungen von Ärztinnen und Ärzten, der Einsatz in der Tierhaltung sowie die Konzentrationen in Wasser- und Bodenproben miteinander korreliert werden. Dadurch lassen sich Rückschlüsse auf die Faktoren der Resistenzbildung ziehen sowie gezielte Gegenmaßnahmen entwerfen. Eine gemeinsame Entwicklung von ML-Verfahren erfordert, dass die Daten aus den einzelnen Sektoren miteinander verknüpft werden, zum Beispiel um resistente Keime in der Umwelt mit denen im Krankenhaus vergleichen zu können. Die abgeleiteten Maßnahmen müssen darüber hinaus sektorübergreifend umgesetzt werden.

Die Bekämpfung von AMR steht in diesem Sinne beispielhaft für die komplexen Gesundheitsgefahren im 21. Jahrhundert und die Notwendigkeit eines ganzheitlichen Gesundheitsansatzes. Essenziell ist in diesem Zusammenhang nicht nur ein ganzheitliches und quantifizierbares Prozessverständnis, sondern auch eine umfassende Nutzbarmachung der verfügbaren Gesundheits- und Umweltdaten. Zusammen bilden beide Komponenten die Grundlage für entsprechende gesellschaftliche Anpassungsmaßnahmen. KI kann und soll an dieser Stelle nicht als alleiniges methodisches Werkzeug dienen. Vielmehr zeigt KI das nötige Potenzial, klassische Statistik, Modellierung, Surveillance- und Visualisierungsmethoden fundamental zu erweitern, um die Vision von One Health umzusetzen.

## Footnotes

1 https://www.who.int/news-room/questions-and-answers/item/one-health und https://www.who.int/teams/one-health-initiative (zugegriffen am 21.04.2023).
